# Ambroxol attenuates detrimental effect of LPS-induced glia-mediated neuroinflammation, oxidative stress, and cognitive dysfunction in mice brain

**DOI:** 10.3389/fimmu.2025.1494114

**Published:** 2025-03-06

**Authors:** Safi Ullah, Tae Ju Park, Jun Sung Park, Abubakar Atiq, Jawad Ali, Min Hwa Kang, Waqar Ali, Kyonghwan Choe, Myeong Ok Kim

**Affiliations:** ^1^ Division of Life Science and Applied Life Science (BK21 FOUR), College of Natural Sciences, Gyeongsang National University, Jinju, Republic of Korea; ^2^ Haemato-oncology/Systems Medicine Group, Paul O’Gorman Leukaemia Research Centre, Institute of Cancer Sciences, College of Medical, Veterinary and Life Sciences (MVLS), University of Glasgow, Glasgow, United Kingdom; ^3^ Department of Psychiatry and Neuropsychology, School for Mental Health and Neuroscience (MHeNs), Maastricht University, Mastricht, Netherlands; ^4^ Alz-Dementia Korea Co., Jinju, Republic of Korea

**Keywords:** lipopolysaccharide, neuroinflammation, glial cells, oxidative stress, cognitive, cognitive impairment, neurodegeneration, ambroxol

## Abstract

Neurodegenerative diseases, such as Alzheimer’s disease (AD) and Parkinson’s disease (PD), are multifactorial. Among various factors, lipopolysaccharides (LPSs) from Gram-negative bacteria, such as *E. coli*, are considered potential causative agents. Despite significant advancements in the field, there is still no cure. In this study, we investigated the neuroprotective effects of ambroxol against LPS-induced neuroinflammation, oxidative stress, neurodegeneration, and the associated cognitive dysfunction. Intraperitoneal injection of LPS (250 µg/kg every alternative day for a total of seven doses over 14 days) triggered glial cell activation, neuroinflammation, oxidative stress, and neurodegeneration in the mouse brain. Ambroxol treatment (30 mg/kg/day for 14 days) significantly reduced neuroinflammation and oxidative stress compared to LPS-treated mice. Immunoblotting and immunofluorescence results showed that ambroxol reduced levels of Toll-like receptor 4 (TLR4) and oxidative stress kinase phospho-c-Jun N-terminal Kinase 1 (p-JNK). It also decreased astrocyte and microglia activation in the cortex and hippocampus of LPS+ Amb-treated mice, as indicated by the downregulation of GFAP and Iba-1. Furthermore, ambroxol-reversed LPS-induced neuroinflammation by inhibiting inflammatory mediators, such as IL-1β and TNF-α, through regulation of the transcription factor p-NFkB. Persistent neuroinflammation disrupted the natural antioxidant mechanisms, leading to oxidative stress. Ambroxol treatment upregulated antioxidant markers, including Nrf-2, HO-1, and SOD, which were downregulated in the LPS-treated group. Additionally, ambroxol-inhibited lipid peroxidation, maintaining malondialdehyde levels in the mouse brain. Ambroxol also improves synaptic integrity by upregulating synaptic biomarkers, including PSD-95 and SNAP-23. Overall, ambroxol demonstrated anti-inflammatory, antioxidant, and neuroprotective effects in LPS-treated mice, highlighting its potential benefits in neurological disorders.

## 1 Introduction

Neuroinflammation is an inflammatory process that contributes to cognitive impairments and neurodegenerative diseases, such as Alzheimer’s disease (AD) and Parkinson’s disease (PD) (1). Glial cells, particularly microglia and astrocytes, are resident macrophages of the central nervous system (CNS) and are majorly involved in triggering and processing neuroinflammation (2). At the level of the CNS, the neuroinflammation may be acute and chronic. Although acute neuroinflammation plays a protective role in the living body, chronic neuroinflammation may disrupt CNS homeostasis, potentially leading to neurodegeneration (3). In a normal physiological state, microglia are responsible for the elimination of harmful metabolic and toxic substances. However, in stress conditions, it may activate and migrate toward the lesion and eliminate the unwanted cellular debris (4). Microglial activation is essential and crucial for the host defense system; however, excessive and prolonged activation of microglia leads to neuronal toxicity and an increase in proinflammatory cytokine levels in both the cortex and hippocampus (5). Different literatures revealed that amyloid beta (Aβ) activates the microglia and induces synthesis and release of free radicals, proinflammatory cytokines, and chemokines, such as nitric oxide (NO), tumor necrosis factor alpha (TNF-α), and interleukin 1 beta (IL-1β), which are hallmarks of AD and PD (6, 7). The brain is enriched with high lipid content and more vulnerable to oxidative stress (8). Due to high lipid content, intense neuroinflammation induces mitochondrial oxidative stress and increased levels of reactive oxygen species as well as lipid peroxidation (LPO) at the level of the brain, followed by neurodegenerative disorders (9, 10).

The exogenous factor, such as lipopolysaccharide (LPS), is one of the integral components of gram-negative bacterial endotoxin and is considered a Toll-like receptor 4 (TLR4) ligand (11). TLR4 is a member of the pattern recognition receptor (PRR) group, a large group that includes both intracellular and extracellular receptor families and senses PAMPs (pathogen-associated molecular patterns) or DAMPs (damage-associated molecular patterns) (12). In the CNS, the TLR4 receptor is expressed on the surface of microglia, and once activated, it is then responsible for the production of proinflammatory cytokines, such as TNF-α, IL-1β, and NO, which are the key regulators of the neuroinflammatory process (13). Furthermore, administration of LPS to the rodents may induce cognitive impairment and alter a variety of behaviors, such as anorexia, decreased locomotion, weight loss, exploratory behavior, increased anxiety, somnolence, and general behavioral depression. Some of the symptoms are considered very familiar to clinically relevant symptoms of neurodegenerative disease in humans (14). As LPS is directly attached to the TLR4 receptor and responsible for the processing of inflammatory events through the mitogen-activated protein kinase group of proteins, such as c-Jun N-terminal kinase (JNK) and nuclear factor Kappa B (NFkB), it triggered its downstream signaling pathways (15, 16). This unique characteristic of LPS has got interest and is considered the most suitable model for glial cells mediated neuroinflammation, oxidative stress, and neurodegeneration-like pathology.

Natural, synthetic, and semisynthetic bioactive substances are used to reduce the morbidity and mortality associated with neurodegeneration. These substances may originate from plants or be isolated from micro- and macro-organisms (17). Among them, plant-based sources have gained significant interest in the prevention and treatment of AD- and PD-like pathologies (18). Ambroxol hydrochloride (2-amino-3, 5-dibromo-*N*-methylbenzylamine hydrochloride) is a natural alkaloid majorly used as a mucolytic agent for asthmatic patients (19). Ambroxol not only has mucolytic properties but also possesses anesthetic, anti‐inflammatory, and antioxidant characteristics (20). It inhibits microglial activation and decreases the accumulation of proinflammatory cytokines (21). According to the literature, ambroxol is being repositioned for diseases like PD, Gaucher’s disease, PD dementia, and dementia with Lewy bodies because it lowers α‐synuclein levels in humans and animal models and increases glucocerebrosidase (GCase) activity (22). Another literature study revealed that ambroxol may attenuate the volume of acute and chronic ischemic stroke by inhibiting the inflammatory processing (23). Herein, we suggested that intraperitoneal (IP) administration of ambroxol may attenuate the LPS-induced neurodegeneration mediated by neuroinflammation and mitochondrial oxidative stress.

## 2 Materials and methods

### 2.1 Chemicals

Ambroxol hydrochloride (CAS 23828-92-4) was purchased from Santa Cruz Biotechnology. LPSs from *Escherichia coli* (*E. coli* O111:B4/L2630.), and all the remaining chemicals used in current study design were acquired from Sigma-Aldrich (St. Louis, MO, USA).

### 2.2 Antibodies

The primary antibodies used in western blot and confocal microscopy in our present study are listed in **Table 1**.

**Table 1:** List of antibodies used in Western blot and immunofluorescences are.

Antibodies	Source	Application	Concentrations	Catalog number	Manufacturer
TLR4	Mouse	WB	1:1000	Sc-293072	Santa Cruz
GFAP	Mouse	WB/IF	1:1000/1:100	sc-33673	Santa Cruz
Iba-1	Mouse	WB	1:1000	17198	Cell Signaling, USA
p-NFkB	Mouse	WB	1:1000	Sc136548	Santa Cruz
P-JNK	Mouse	WB	1:1000	Sc-6254	Santa Cruz
TNF-α	Mouse	WB	1:1000	sc-52746	Santa Cruz
IL-1β	Mouse	WB	1:1000	sc-32294	Santa Cruz
Nrf2	Mouse	WB/IF	1:1000/1:100	12721S	Cell Signaling, USA
HO-1	Mouse	WB	1:1000	sc-136961	Santa Cruz
PSD-95	Mouse	WB	1:1000/1:100	sc-71933	Santa Cruz
SNAP-23	Mouse	WB	1:1000	sc-374215	Santa Cruz

The secondary antibodies (anti-mouse and anti-rabbit) were diluted with 1:10,000 in 1X TBST.

### 2.3 Experimental animals

Wild-type C57BL/6N male 8-week-old mice (*n* = 32, with eight mice per group) weighing between 26 and 28 g were acquired from Samtako Bio, Osan, South Korea. First, the mice were acclimatized for one week, keeping control of temperature and humidity (23°C ± 2°C and 60 ± 15%, respectively) with a regulated 12-h light/12-h dark cycle. All the mice were allowed to freely eat standard pellet food and drink water ad libitum. All experimental procedures were performed keeping the standardization of the Institutional Animal Care and Use Committee (IACUC) of the Division of Applied Life Sciences, Department of Biology, at Gyeongsang National University, South Korea.

### 2.4 Mice groupings and drug administration

**Figure 1:**
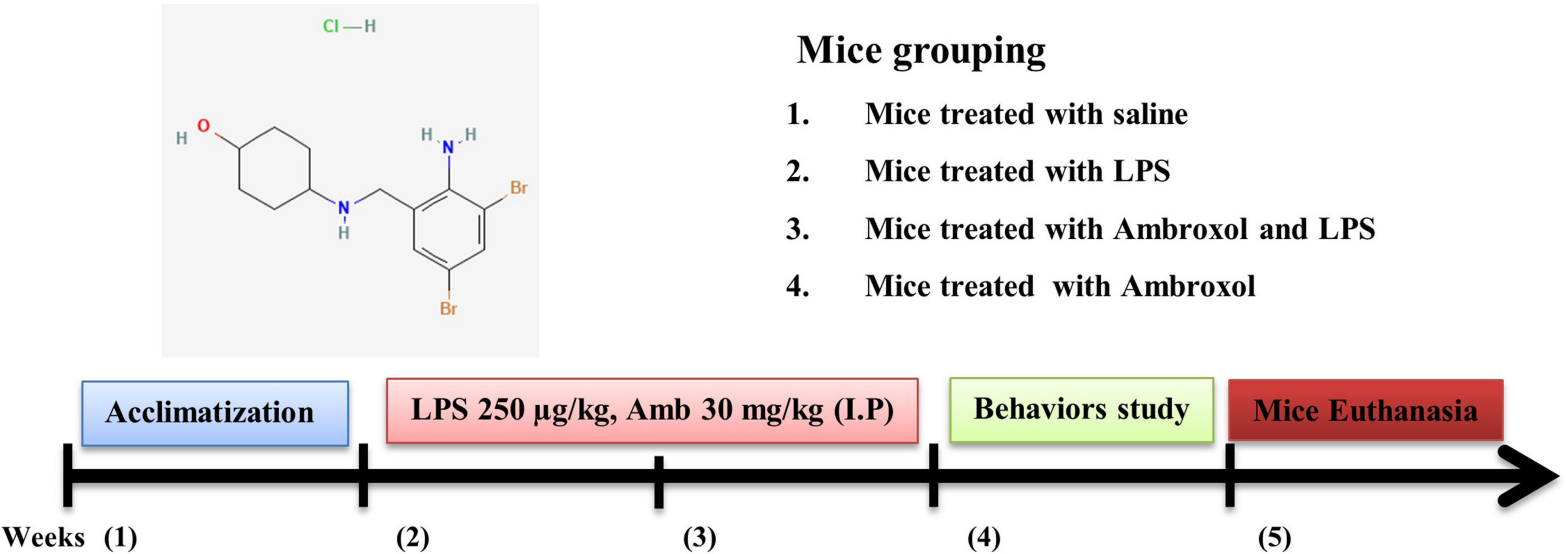
A schematic representation of the experimental designed to investigate the effectiveness of ambroxol against LPS-induced neuroinflammation, oxidative stress, and memory impairments.

All the mice were randomly divided into four distinct group: (a) a control group treated with 0.9% normal saline; (b) LPS-injected group (LPS 250 µg/kg IP every alternative day for 2 weeks); (c) LPS + Ambroxol co-treated group (LPS 250 µg/kg and Ambroxol 30 mg/kg, IP); for 14 days (Ambroxol was administered simultaneously with LPS); (d) Only Ambroxol treated group (30 mg/kg for 2 weeks consecutively). Both the LPS and Ambroxol was dissolved in sufficient amount of normal saline and administered IP route (**Figure 1**).

### 2.5 Behavioral tests

The Morris water maze (MWM) test was performed as discussed previously with little modification (24). The MWM was used to measure declarative learning and memory in laboratory rodents. This setup consists of a circular water tank (width 100 cm, height 40 cm) having four different quadrants and filled with water dissolved in non-toxic white ink. A hidden platform (4.5 cm in diameter and 14.5 cm in height) was placed slightly below the surface of water in specific quadrants. The mice were freely allowed to move in order to locate the hidden platform. All the mice were individually receive four training trials for five consecutive days. On the fifth day, the hidden platform was removed, allowing the mice to swim freely for 1 min. The time spent by each mouse in the targeted quadrant and the number of crossings of over the location of the hidden platform were measured. All the procedures were recorded on the video graphic camera attached above. The data were analyzed by motion tracking software (SMART 3.0, Panlab Harvard Apparatus, Bioscience Company, and Holliston, MA, USA).

To explore spontaneous alteration behavioral changes and exploratory activity, the Y-Maze test was performed as previously reported (25). The Y-maze assembly consisted of three equal arms (each arm is 50 cm in length, 10 cm in width, and 20 cm in height). All the mice were placed in the center of the Y-maze assembly one by one, allowing movement with an interval of 8 min. All arm entries and alterations were recorded with the motion sensor attached above. The spontaneous alteration percentage (%) was calculated as (entries into three different arms consecutively/total number of arm entries-2) × 100. The increase in percentage of spontaneous behavior reflects the cognitive and memory dysfunction and vice versa.

### 2.6 Extraction of proteins from brain tissues

Post-behavioral analysis, all the mice were sacrificed, and both the cortexes and hippocampal tissue were isolated as described previously (8). The cortical and hippocampal tissues were then homogenized using PRO-PREP (iNtRON Biotechnology, Dallas, Texas, MA, USA) solution. After that, the samples were centrifuged for a duration of 25 min at 4°C with a speed of 13,000 revolutions per minute (r.p.m.). The supernatants were collected and preserved at −80°C.

### 2.7 Western blot analysis

The protein concentrations were measured by using the BioRad Kit as reposted previously (26). Equal amounts of protein were loaded in 12%/10% (depending upon the molecular weight of protein) sodium dodecyl sulfate–poly-acrylamide gels and then transferred to polyvinylidene fluoride (PVDF) membranes (Immobilon-PSQ, Transfer membrane, Merck Millipore, Burlington, MA, USA). The PVDF membranes were blocked with 5% skim milk w/v dissolved in 1X TBST solution and incubated with primary antibodies overnight at 4°C. On the next day, the membranes were washed with a 1X TBST solution and again incubated with the secondary antibodies for a duration of 1 to 2h (either anti-mouse/anti-rabbit depending on the primary source of the antibody). Furthermore, it was washed with 1X TBST, and to detect the protein level, the ECL fluorescence reagent (EzWestLumiOne, ATTO, Tokyo, Japan) was used. The x-ray films were quantified using ImageJ software (v. 1.50, NIH, Bethesda, MD, USA). Whereas β-actin normalizes the expression levels of the protein of interest, guarantees uniform protein loading and transfer across lanes, and acts as an internal reference to take experimental condition heterogeneity into account.

### 2.8 Preparation of brain sections for morphological analysis

All the mice for the confocal study were perfused transcardially with normal saline (0.9%) following 4% paraformaldehyde for a duration of 4 min. The brain was isolated from each mouse fixed with 4% paraformaldehyde for 72h. Similarly, all the brains were kept in 20% sucrose for a duration of 72h. Now, the brain was blocked with optimal cutting temperature compound (O.C.T. compound) (Sakura Finetek USA, Inc., Torrance, CA, USA) and stored at −80°C. The brain was sliced (14 µm) using a CM3050C cryostat (Leica, Germany). All the brain sections were thaw-mounted on gelatin-coated slides (Fisher, Rockford, IL, USA) and stored again at −80°C.

### 2.9 Immunofluorescence staining for confocal laser microscopy

Immunofluorescence analysis was performed as described previously (27). The slides were washed twice with filtered phosphate buffer saline (PBS, 1%) for 10 min at room temperature. Apply the proteinase K solution for 5 min and wash again with 1% PBS. The slides were then incubated for 1h with a specific solution consisting of 2% normal goat serum and 0.3% Triton X-100 dissolved in 1% PBS. The slides were then incubated with required antibodies (diluted in PBS, 1:100) overnight and kept at 4°C. On the next day, the slides were washed and incubated with secondary antibodies for 2h. The nuclear staining dye 4′, 6-diamidino-2-phenylindole was applied for 10 min. Now, the slides were dried, a few drops of mounting media (DAKO) were put on, and they were covered with coverslips. The slides were ready to observe under confocal laser microscopes, and the images were quantified by using ImageJ software. All the images were graphically and statistically analyzed by GraphPad Prism software (ver. 8.0, San Diego, CA, USA) (28).

### 2.10 Assessment of antioxidant enzyme SOD and oxidative stress marker MDA

Investigations were conducted on the impact of ambroxol treatment on SOD activity and malondialdehyde (MDA), which is the oxidative stress marker. The SOD assay was carried out according to previous studies (29). In summary, the 96 well plates (200 μl reaction mixture) were filled with a mixture of tris-EDTA (50 mM, pH 8.5), pyragallol (24 mM), and sample. The absorbance was recorded at 420 nm. Each activity was conducted three times independently. Additionally, the LPO rate was calculated using modified Utley et al. protocols by calculating the MDA concentration. The assay mixture is made up of 200 μl supernatant, 20 μl mM ferric chloride, 200 μl 100 mM ascorbic acid, and 580 μl of 0.1 mM phosphate buffer (pH 7.4). It is then incubated for 60 min at 37°C in a water bath. Following 1h of incubation, samples were treated with 1000 μl of 10% trichloroacetic acid and 1000 μl of 0.66% thiobarbituric acid (TBA) to stop the reaction. The tubes were kept in the water bath for 20 min, chilled in an ice bath, and then centrifuged for a 10-min period at 3000 × g. The concentration of thiobarbituric acid reactive substances (TBARS) was determined at 535 nm utilizing supernatant absorbance and a blank solution containing all reagents except the test sample. The result was expressed as nM TBARS/min/mg protein.

### 2.11 Statistics

The data were analyzed through ImageJ software in order to measure the density, which is expressed as an arbitrary unit (AU) for both morphological and Western blot analyses. All the data were shown in mean ± standard error mean (SEM) of eight mice per group. All the groups are compared with each other by using one-way analysis of variance with Tukey’s *post hoc* test. *P*-values less than 0.05 were considered to be statistically significant. The symbol **#** indicates a significant difference from the saline-injected group, while the symbol * indicates a significant difference from the LPS-injected group. Significance: **^#^** *p* ≤ 0.05, **^##^** *p* ≤ 0.01, and **^###^** *p* ≤ 0.001; *p ≤ 0.05, **p ≤ 0.01, and ***p ≤ 0.001.

## 3 Results

### 3.1 Ambroxol regulates the expression of TLR4, glial cells, and its downstream targets

**Figure 2:**
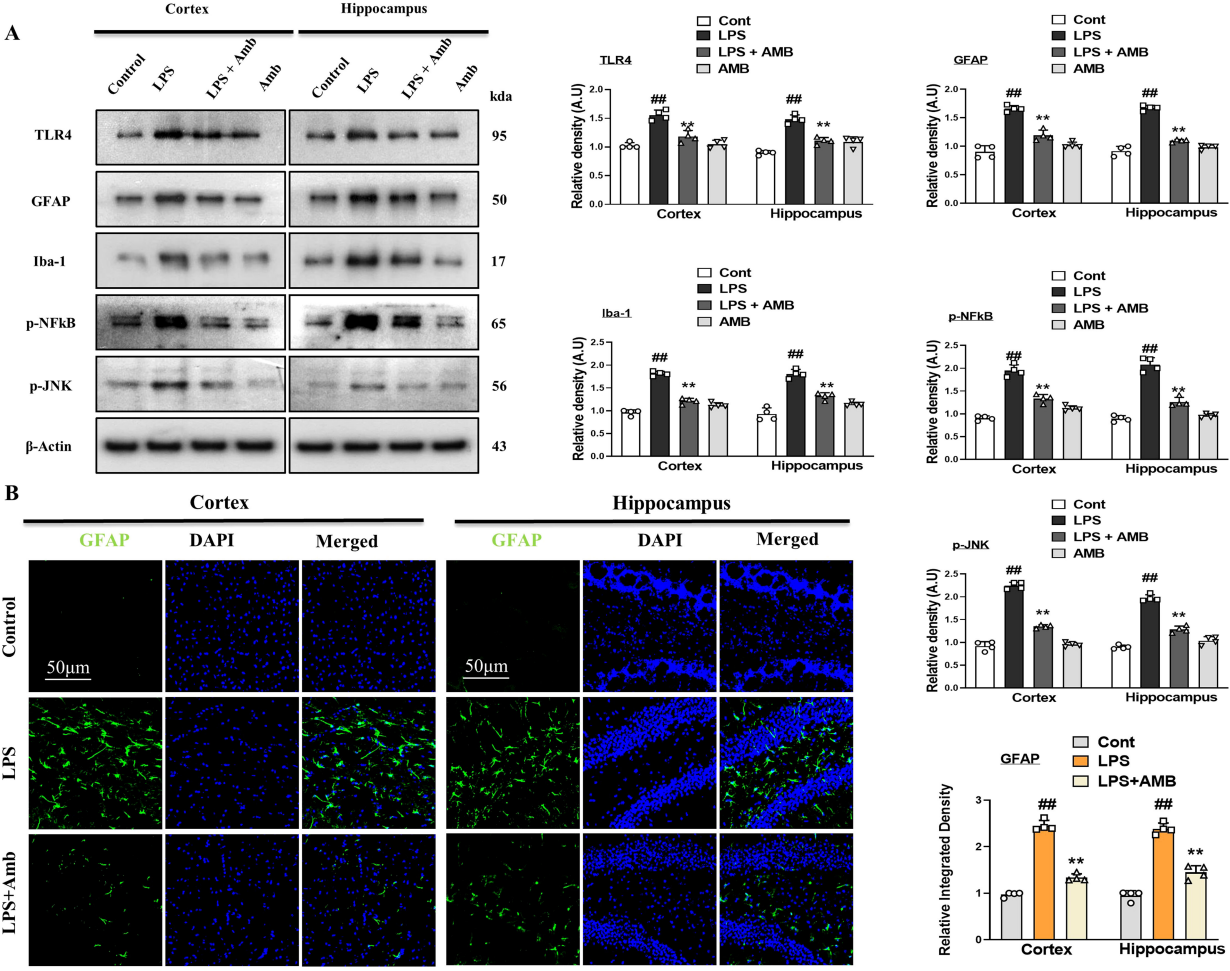
Ambroxol regulates neuroinflammation in the LPS-induced mice. **(A)** Showing Immunoblot results with respective histograms of TLR4, GFAP, Iba-1, p-NFkB, and p-JNK mice brains (*n* = 4). **(B)** Immunofluorescence images of GFAP with respective graphs describe relative integrated density for the cortex and hippocampus (DG) in the LPS-treated mice brain (n=4). Beta-actin was used as a loading control. All the data were measured in mean ± S.E.M. ^#^significantly different from the saline-injected group, *significantly different from the LPS-injected group. Significance: ^#^ *p* ≤ 0.05, ^##^ *p* ≤ 0.01 and ^###^ *p* ≤ 0.001; *p ≤ 0.05, **p ≤ 0.01 and ***p ≤ 0.001.

Previous literature has shown that there is enhanced expression of TLR4 with LPS, due to which activation of astrocytes and microglia occurs (30). To analyze the effects of LPS on TLR4 and its downstream inflammatory markers in the mouse cortex and hippocampus, we performed Western blotting for TLR4, Iba-1, GFAP, p-NFkB, and p-JNK. According to our results, there was increased expression of the above-mentioned inflammatory biomarkers with LPS administration, which was significantly downregulated in the LPS+Amb co-treated group (**Figure 2A**). These beneficial effects were further confirmed by fluorescent microscopy, using GFAP antibody, showing elevated immunoreactivity in the cortical and hippocampal regions in the LPS–injected groups that were significantly attenuated with the LPS+Amb group. Both WB and IF results suggest that Ambroxol may inhibit the upregulation of TLR4 and p-NFkB, which may aid in the neuroprotective mechanisms of Ambroxol (**Figure 2B**).

### 3.2 Ambroxol abrogates LPS-induced TNF-α and IL-1β level in mice brain

**Figure 3:**
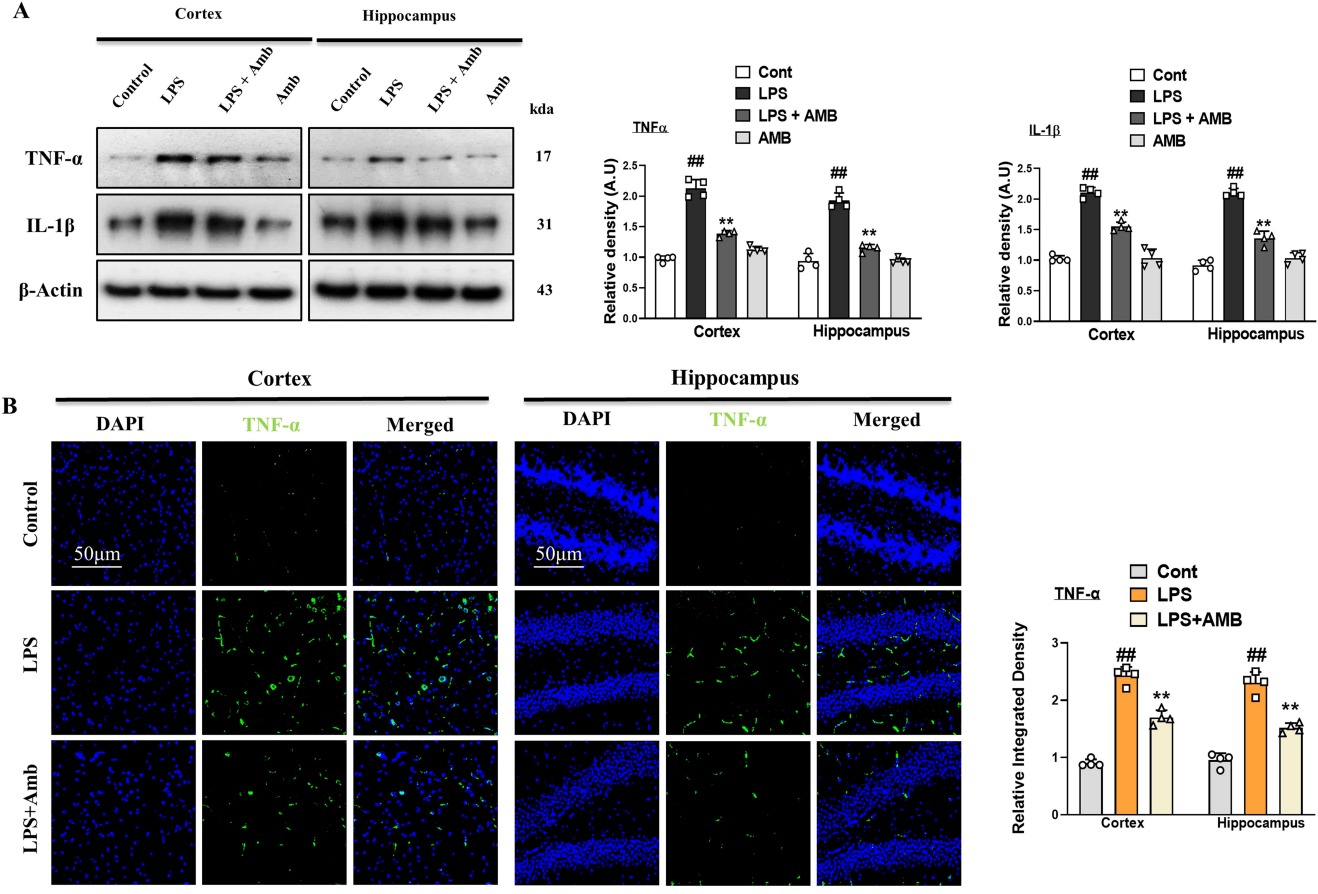
Ambroxol regulates the expression of inflammatory cytokines. **(A)** Immunoblot analysis of TNF-α and IL-1β in the experimental groups, accompanied by corresponding bar graphs (*n* = 4). **(B)** Immunoreactivity of TNF-α in the cortex and hippocampus (*n* = 4), as illustrated by immunofluorescence photographs with corresponding bar graphs scale bar 50 µm. All data were expressed as mean ± S.E.M. ^#^significantly different from the saline-injected group, *significantly different from the LPS-injected group. Significance: ^#^ *p* ≤ 0.05, ^##^ *p* ≤ 0.01, and ^###^ *p* ≤ 0.001; *p ≤ 0.05, **p ≤ 0.01, and ***p ≤ 0.001.

Glial cell activation leads to the synthesis and secretion of TNF-α and interleukin-1β (IL-1β) and takes part in inflammatory diseases. To investigate the level of TNF- α and IL-1β we performed western blotting and immunofluorescences. Our western blot result revealed that both the levels of TNF- α and IL-1β were increased in the LPS-only subjected group. Similarly, this elevated level of TNF- α and IL-1β was remarkably decreased in the LPS+Amb co-treated group (**Figure 3A**). Additionally, we performed the immunofluorescences of brain tissues of different groups of experimental animals. The LPS-treated group showed high reactivity of TNF- α as compared to the controlled group. Whereas the LPS+Amb treated group showed less immunoreactivity as compared to the LPS-only treated group, as shown in **Figure 3B**.

### 3.3 Ambroxol inhibit oxidative stress by regulating the level of NRF2, HO-1, SOD, and MDA in mice brain

**Figure 4:**
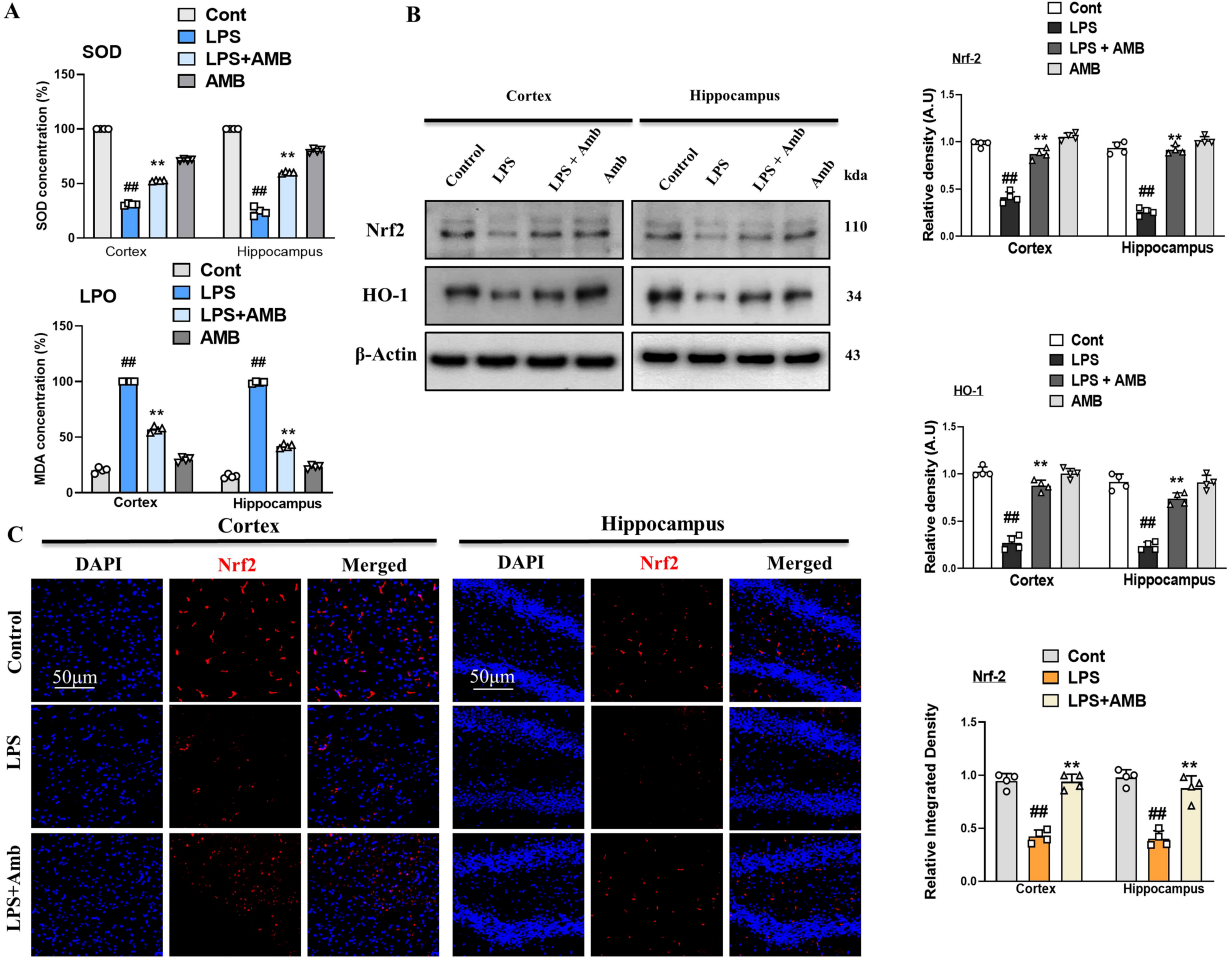
Ambroxol mitigates oxidative stress induced by LPS treatment. **(A)** Representing SOD and LPO assay respectively. **(B)** Immunoblot analysis and bar graphs of the expression of the proteins Nrf2 and HO-1 in the mouse cortex and hippocampus (*n* = 4). **(C)** Immunofluorescence images showing of Nrf2 in the experimental group, with a scale bar of 50 µm. All data were presented as mean ± S.E.M. (*n* = 4). Significant differences were seen between different experimental groups. ^#^significantly different from the saline-injected group, *significantly different from the LPS-injected group. Significance: ^#^ *p* ≤ 0.05, ^##^ *p* ≤ 0.01 and ^###^ *p* ≤ 0.001; *p ≤ 0.05, **p ≤ 0.01, and ***p ≤ 0.001.

Neuroinflammation leads to elevated oxidative stress and downregulates the level of antioxidant protein (NRF2 and HO-1) (31). The antioxidant activity of SOD in cortical and hippocampal tissues was significantly reduced following LPS injection. Ambroxol significantly alleviated this reduction. Furthermore, cells experience severe stress when phospholipid peroxidation leads to the production of MDA. The MDA levels were significantly elevated in the LPS group compared to the normal control group (*p* < 0.001). The attenuative potential of Ambroxol was further supported by the significant reduction (*p* < 0.001) in MDA levels, highlighting its effective role in mitigating oxidative stress (**Figure 4A**). Similarly, we assessed the concentrations of nuclear factor erythroid 2-related factor 2 (NRF2) and its subsequent antioxidant enzyme, oxygenase-1 (HO-1), in the cortical and hippocampal tissues of mice. Our Western blot result revealed decreased expression of NRF2 and HO-1 in LPS-injected mice as compared to the normal control, whereas Ambroxol attenuated the NRF2 and HO-1 expression caused by LPS in the brain tissue of the LPS-Amb co-treated mice. These results show that Ambroxol mitigated the level of oxidative stress by regulating the NRF2 and HO-1 expression level in the cortical and hippocampal tissues induced by LPS administration (**Figure 4B**). Additionally, in order to reinforce the western blot results, we performed the immunofluorescence analysis, which confirmed that the LPS-injected mice showed decreased immunoreactivity of NRF2, while the LPS+Amb co-treated group significantly reversed these effects and increased immunofluorescence reactivity of NRF2 in the cortex and hippocampus, suggesting its antioxidant potential (**Figure 4C**).

### 3.4 Ambroxol reversed LPS-induced synaptic dysfunction by regulating synaptic markers in the hippocampus and cortex regions

**Figure 5:**
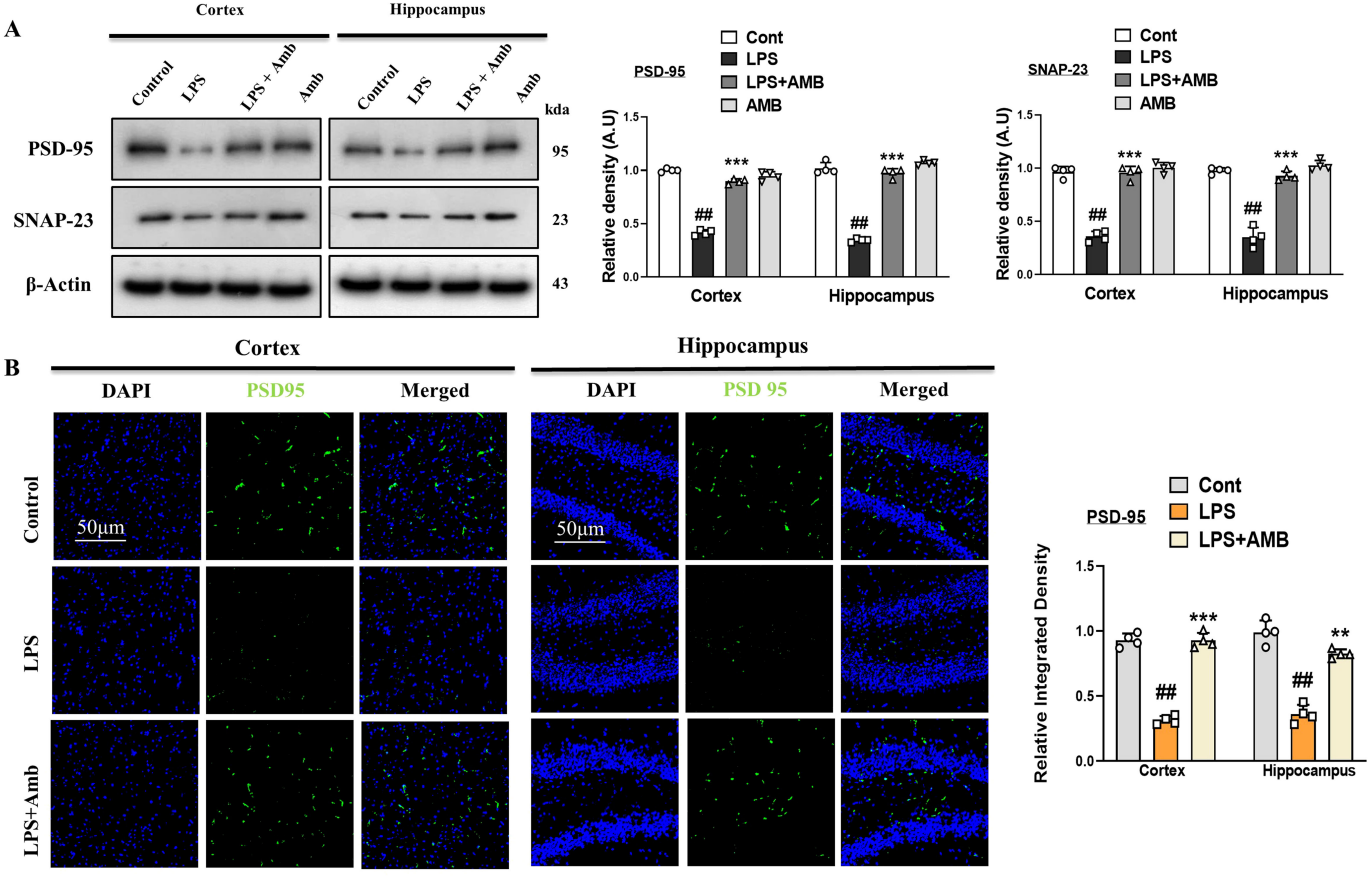
Ambroxol regulated synaptic integrity. **(A)** Western blots analysis of PSD-95 and SNAP-23 in the cortex and hippocampus of mice brain (*n* = 4). **(B)** Immunofluorescence images and bar graphs of PSD-95 in the cortical and hippocampal regions of the mouse brain (*n* = 4), with a scale bar of 50 µm. All data were expressed as mean ± S.E.M. ^#^significantly different from saline-injected group, *significantly different from LPS-injected group. Significance: ^#^ *p* ≤ 0.05, ^##^ *p* ≤ 0.01, and ^###^ *p* ≤ 0.001; *p ≤ 0.05, **p ≤ 0.01, and ***p ≤ 0.001.

LPS-induced neuroinflammation and elevated oxidative stress collectively leads to neuronal toxicity and synaptic dysfunction. To measure integrity of synapsis we measured level of both pre and post synaptic biomarkers such as SNAP-23 and PSD-95 respectively. The Western blot results showed a reduced in expression of SNAP-23 and PSD-95 in the LPS-treated group as compared to the normal saline-injected group. Notably, the expressions of these synaptic biomarkers were upregulated in the LPS+Amb-cotreated group (**Figure 5A**). These effects of ambroxol were further confirmed by performing immunofluorescence of PSD-95 in cortex and hippocampus of different mice group. Our findings, suggest that PSD-95 expression was reduced in LPS-treated mouse brains compared to the saline-injected control group. Notably, the immunofluorescence of the PSD-95 was markedly increased in the LPS + Amb-cotreated group as shown in **Figure 5B**.

### 3.5 Ambroxol improved LPS-induced behavioral and cognitive deficits

**Figure 6:**
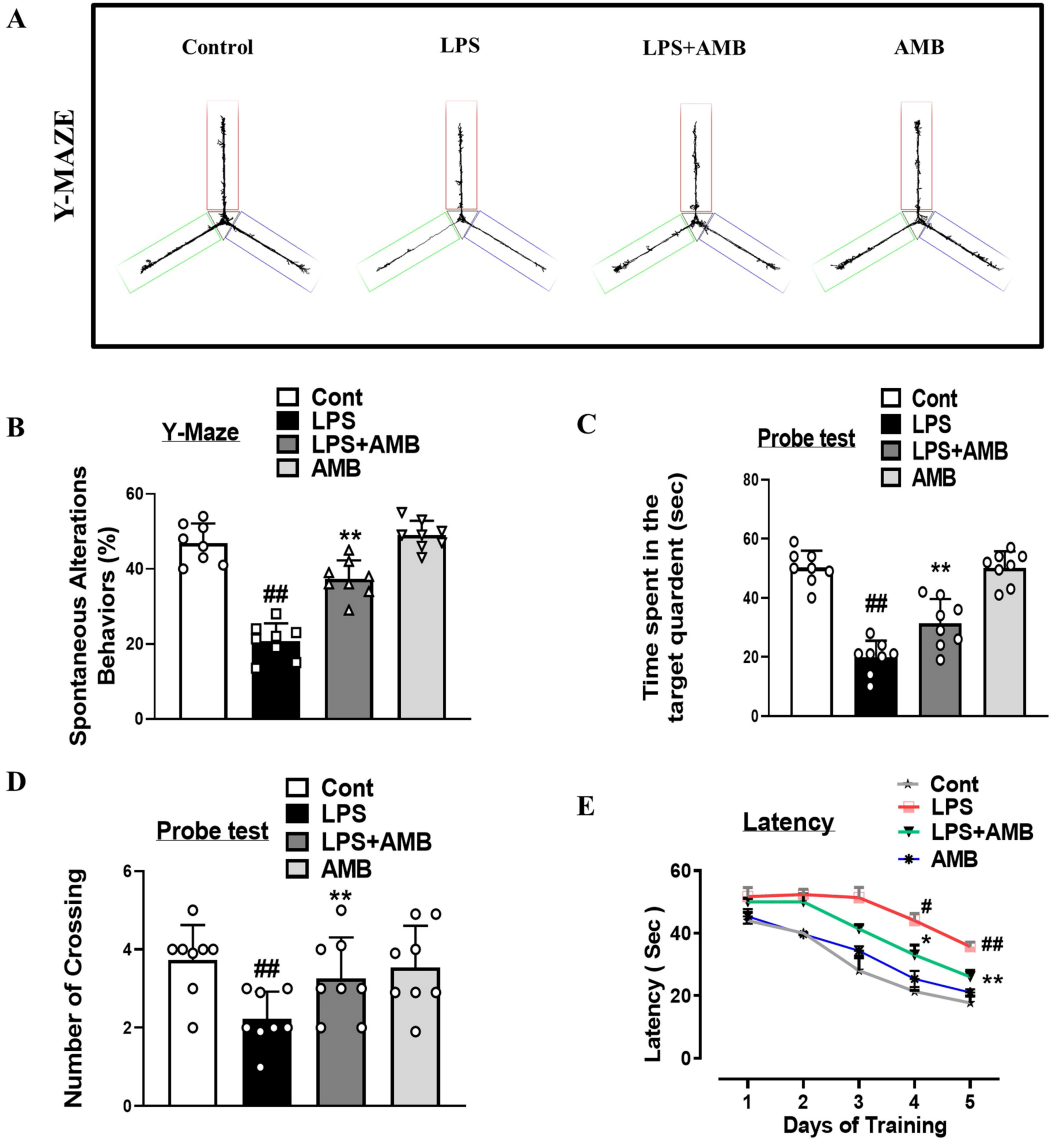
Ambroxol improves LPS-induced memory and cognitive dysfunction. **(A)** Visuals of the trajectory paths in the Y-maze tasks. **(B)** Percentage of spontaneous alternation behavior and Y-maze analysis. **(C)** Time spent in the targeted quadrant **(D)** Number of crossings over the hidden platform in MWM. **(E)** Line graph describing the average time taken to reach the platform in targeted quadrant. Mean ± S.E.M. was used to measure all the results (*n* = 8 per group). ^#^significantly different from saline-injected group, *significantly different from LPS-injected group. Significance: ^#^ *p* ≤ 0.05, ^##^ *p* ≤ 0.01, and ^###^ *p* ≤ 0.001; *p ≤ 0.05, **p ≤ 0.01, and ***p ≤ 0.001.

To examine the effects of ambroxol on learning and memory dysfunctions in LPS-injected mice, we performed the Y-maze tests and the MWM. First, we performed the Y-maze test, which showed that LPS leads to short-term special memory impairment. Interestingly, ambroxol treatment increased the percentage of spontaneous alternation behavior, which enhanced spatial working memory function (**Figures 6A, B**). In MWM, after the initial training, the animals were allowed to find the hidden platform, and the latency time was recorded in the MWM task. Our result showed that the LPS-injected mice spent more time in the target quadrant, while LPS+Amb-treated mice took less time to reach the target quadrant where the hidden platform was placed (**Figures 6C–E**). All of these results showed that ambroxol treatment improved learning and memory impairments in the LPS-injected animal model.

## 4 Discussion

Neuroinflammation can be described as an inflammatory response of the CNS to various endogenous and exogenous factors that act against homeostasis. Different types of cells within the CNS response to these factors, such as astrocytes and microglia (32). It has been observed that neuroinflammation together with oxidative stress are fundamental aspects that need to be taken into consideration regarding the onset and progression of neurodegenerative disorders (33). Neuroinflammation is actively involved in neurological diseases and disorders such as AD and PD (34). It is a very complex response involving a host of cellular and molecular changes, recruitment of peripheral immune cells, induction of some intracellular signaling pathways, and release of inflammatory mediators at the level of the brain (35).

Herein, we investigated the neuroprotective mechanism of Ambroxol in the LPS-mouse model, which suggested that Ambroxol suppressed the elevated neuroinflammation and oxidative stress-induced memory impairment and synaptic dysfunctions. LPS was reported to trigger the immune response and inflammation by activating the TLR4 surface receptors (36). It triggers the immune system and also stimulates the process of inflammation in the brain (37). Several lines of investigation demonstrated that LPS-evoked glial cells and macrophages contribute to the synthesis and release of inflammatory mediators followed by ROS generation (38). As a result, there is protein modification, alteration in cell function, and exaggerated systemic inflammatory response (39).

Similarly, in the present study, we observed that LPS evokes inflammatory responses and elevated oxidative stress which, further exhibits harmful effects on neuronal cells (40, 41). Endotoxin, an integral part of the bacterial cell wall, is isolated and known as LPS and injected into mice. The activation of TLR4 by the administration of LPS leads to the phosphorylation of NF-kB and increases the expression of inflammatory biomarkers (42). Previously, it was investigated that LPS exposure significantly activates the glial cells (astrocytes and microglia) in a TLR4-dependent manner (43, 44). The application of natural bioactive substances is a primary interest for the prevention and treatment of neurodegenerative diseases. Ambroxol is one of them, which significantly reduced the activated gliosis via suppression of TLR4. Here, we also investigated that LPS activated the gliosis and significantly increased the GFAP and Iba-1, which was elevated by the Ambroxol treatment in the cortex and hippocampus of the mouse brain (**Figures 2A, B**). Similarly, increased levels of phosphorylated JNK (p-JNK), phosphorylated NF-κB (p-NFκB), and other inflammatory mediators such as tumor necrosis factor-alpha (TNF-α) and IL-1β were observed in the cortex and hippocampus of LPS-treated mouse brains (45). Meanwhile, the expression levels of these inflammatory biomarkers were significantly reduced in the group of mice treated with LPS and Amb (**Figures 2A**, **3A, B**). Additionally, LPS activates other signaling pathways, including the p-JNK signaling pathway, which influences the expression of various genes such as IL-6, TNF-α, and COX-2 (46). Numerous studies have emphasized the critical role of the p-JNK signaling pathway in neurodegenerative diseases due to its involvement in stress-induced responses, apoptosis, neuroinflammation, mitochondrial oxidative bursts, gene regulation, and Tau protein phosphorylation (47).

Previous research has reported that inhibiting TLR4 can reduce the expression and production of IL-1β and TNF-α in the LPS-treated microglial cells (48). Notably, our Western blot and confocal microscopy results also showed that Ambroxol treatment significantly reduced the expression level of the TLR4 in the cortex and hippocampus of the adult mouse brain (**Figures 3A, B**).

Oxidative stress is another parameter and considered a major event in neurodegenerative diseases, multiple sclerosis, and liver cirrhosis (49). Other studies suggested that there is a transcription factor that regulates oxidative stress, such as Nrf2 (50). As a transcription factor, Nrf2 is involved in the regulation of other antioxidant genes, such as HO-1 (51). Furthermore, LPS is responsible for inducing oxidative stress, which reduces the expression of antioxidant protein HO-1 by interfering with the Nrf2 signaling pathway (52). Notably, our Western blot and immunofluorescence results revealed that the levels of Nrf2 and HO-1 were significantly downregulated in the LPS-injected group. Interestingly, in the LPS + Amb co-treated groups, these elevations were remarkably reversed, showing the possible antioxidant effects of ambroxol (**Figures 4A, B**). Antioxidants such as glutathione peroxidase, catalase, and superoxide dismutase were also shown to have lower levels following LPS treatment (53). In addition, our present study determined that after injection of LPS, there was a considerable decrease in the levels of the SOD in the brain cortical and hippocampus tissue, while Ambroxol treatment significantly increased the level of SOD. Moreover, LPO in the form of MDA causes extreme stress in neuronal cells, which was markedly elevated in the LPS group relative to the normal control group. The significant reduction in MDA levels highlighted the protective capability of Ambroxol and highlighted its efficacious role in reducing oxidative stress.

Synapsis play a crucial role in neuronal communication. Any abnormality or structural deformation due to endogenous or exogenous stress may lead to neurological conditions, such as AD and PD (54). Synaptic proteins are responsible for the exocytosis and endocytosis of various neurotransmitters and have been found to be altered in neurodegenerative disorders. Synaptic dysfunction is a common hallmark of dementia, involving both specific presynaptic as well as postsynaptic proteins, which are postsynaptic density protein (PSD-95) and synaptosomal-associated protein 23 (SNAP-23). Previous studies have reported that LPS downregulate the expression levels of SNAP-23 and PSD-95, synaptic proteins that are important for cognitive functions and synaptic plasticity (55). Current data showed that the IP LPS injection significantly downregulated the levels of PSD-95 and SNAP-23; however, the administration of Ambroxol notably reversed the decline in the synapses and increased the levels of these proteins (**Figures 5A, B**). Similarly, from the behavioral study, we examined that Ambroxol treatment significantly reduces the latency time in LPS-treated mice and also increases the time spent in the target quadrant and the number of platform crossings during the probe test of MWM. In addition, during the Y-maze test, we found that the Ambroxol treatment enhanced the % spontaneous alteration behavior. Collectively, the results of MWM and the Y-maze provide the evidence that Ambroxol improved the memory impairments and spatial learning in the LPS-treated mice (**Figures 6A–E**).

## 5 Conclusion

**Figure 7:**
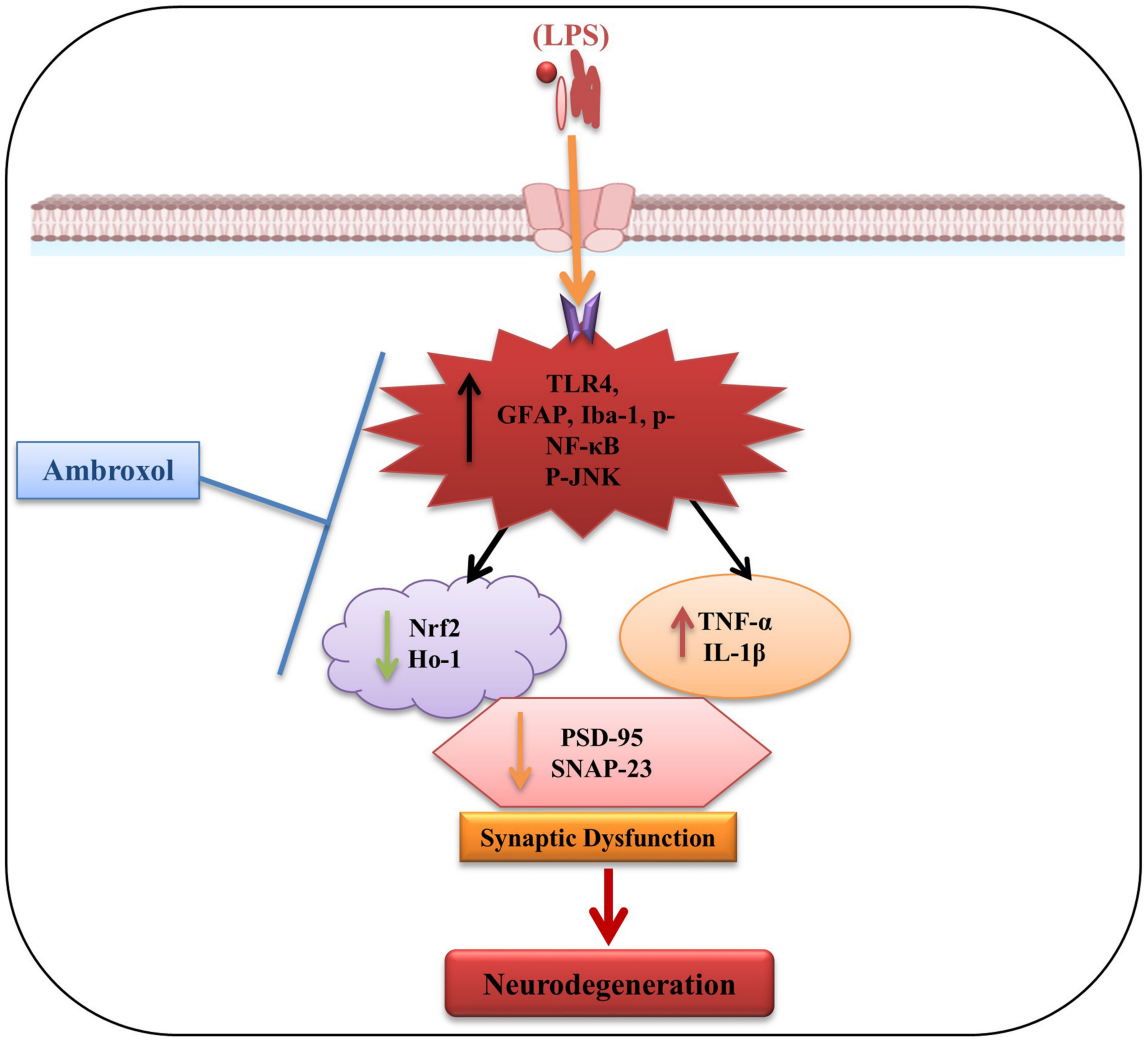
The possible neuroprotective mechanism of Ambroxol’s against LPS-induced neuroinflammation, oxidative stress, and cognitive impairment in the mouse brain.

It is concluded that ambroxol-attenuated LPS-induced neuroinflammation via the TLR4/NF-κB pathway and reduced oxidative stress through Nrf2/HO-1 regulation in C57BL/6N male mice. Our current data strongly showed a good safety profile and a promising new candidate for treating neurodegenerative disorders. Collectively, our results revealed that Ambroxol might be considered an excellent therapeutic drug in LPS-induced memory impairments and neurodegeneration (**Figure 7**).

## Data Availability

The original contributions presented in the study are included in the article/**Supplementary Material**; further inquiries can be directed to the corresponding authors.
