# Supplementary material for: Ambroxol attenuates detrimental effect of LPS-induced glia-mediated neuroinflammation, oxidative stress, and cognitive dysfunction in mice brain
Source: Front Immunol. 2025 Mar 6;16:1494114. doi: 10.3389/fimmu.2025.1494114 (PMC11923628; doi:10.3389/fimmu.2025.1494114)
Supplement: Supplementary file 2 [file Presentation1.pptx]

## Slide 1
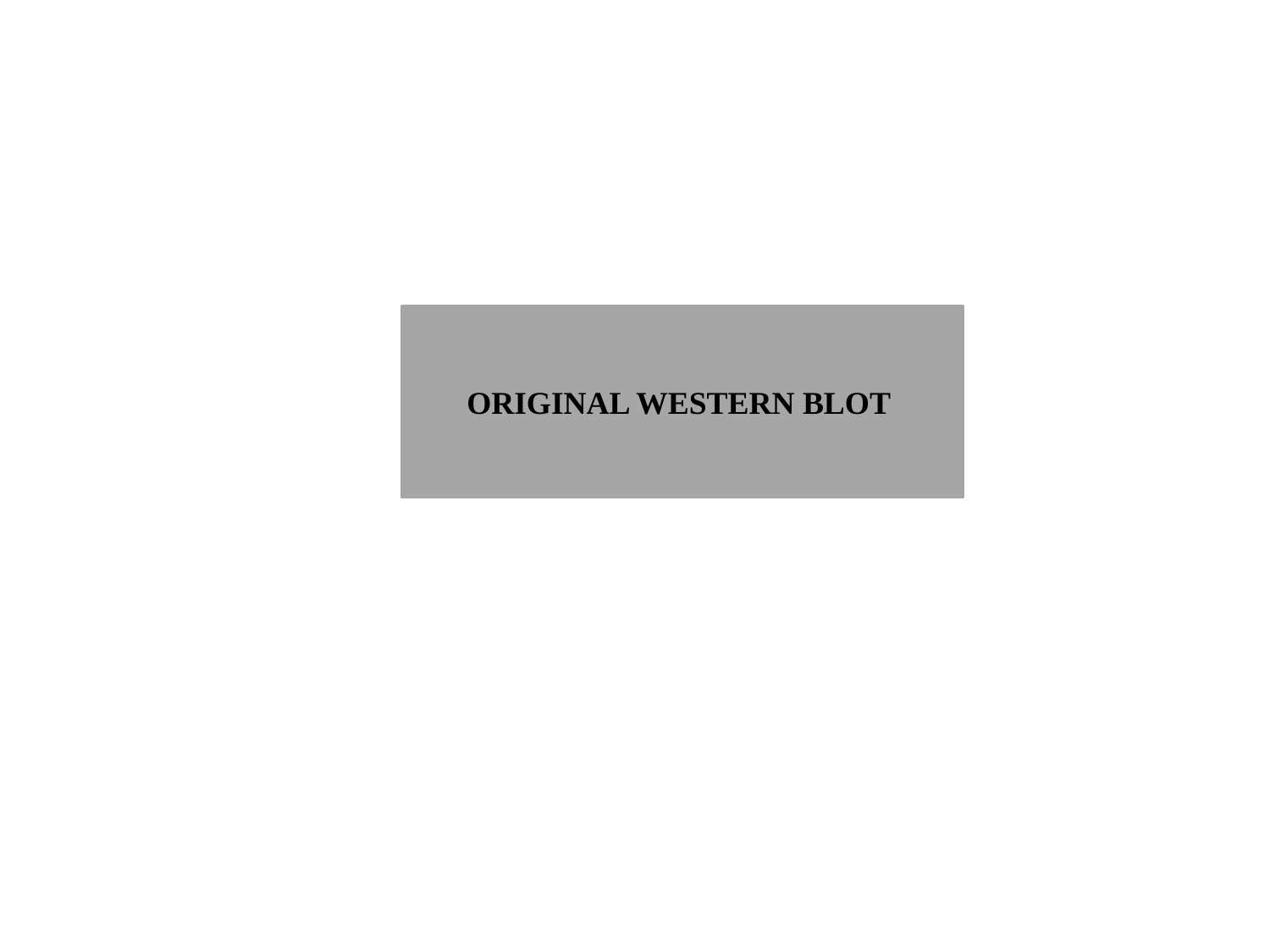

ORIGINAL WESTERN BLOT

## Slide 2
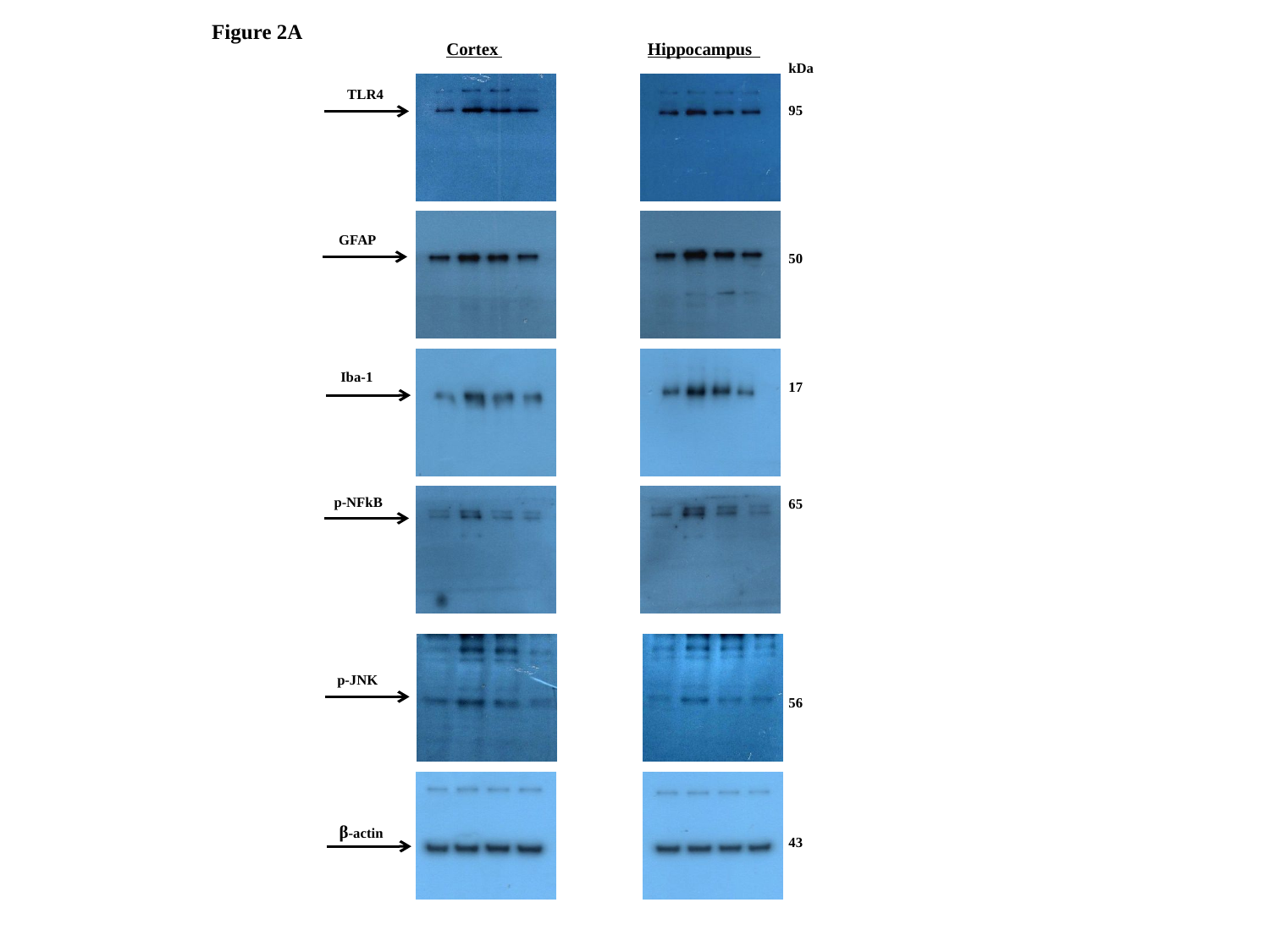

Figure 2A
Cortex
Hippocampus
kDa
TLR4
95
GFAP
50
Iba-1
17
p-NFkB
65
p-JNK
56
β-actin
43

## Slide 3
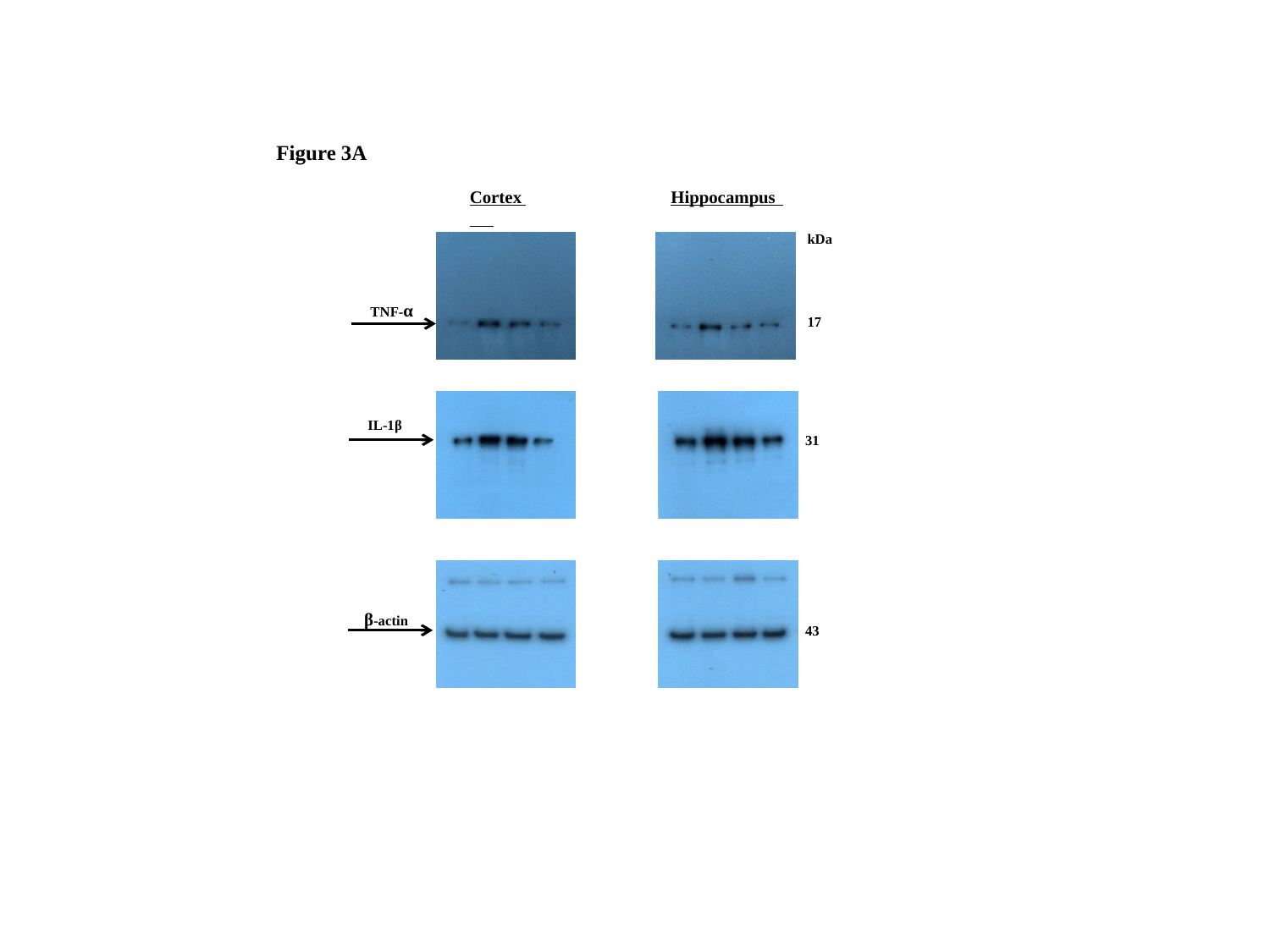

Figure 3A
Cortex
Hippocampus
kDa
 TNF-α
17
 IL-1β
31
 β-actin
43

## Slide 4
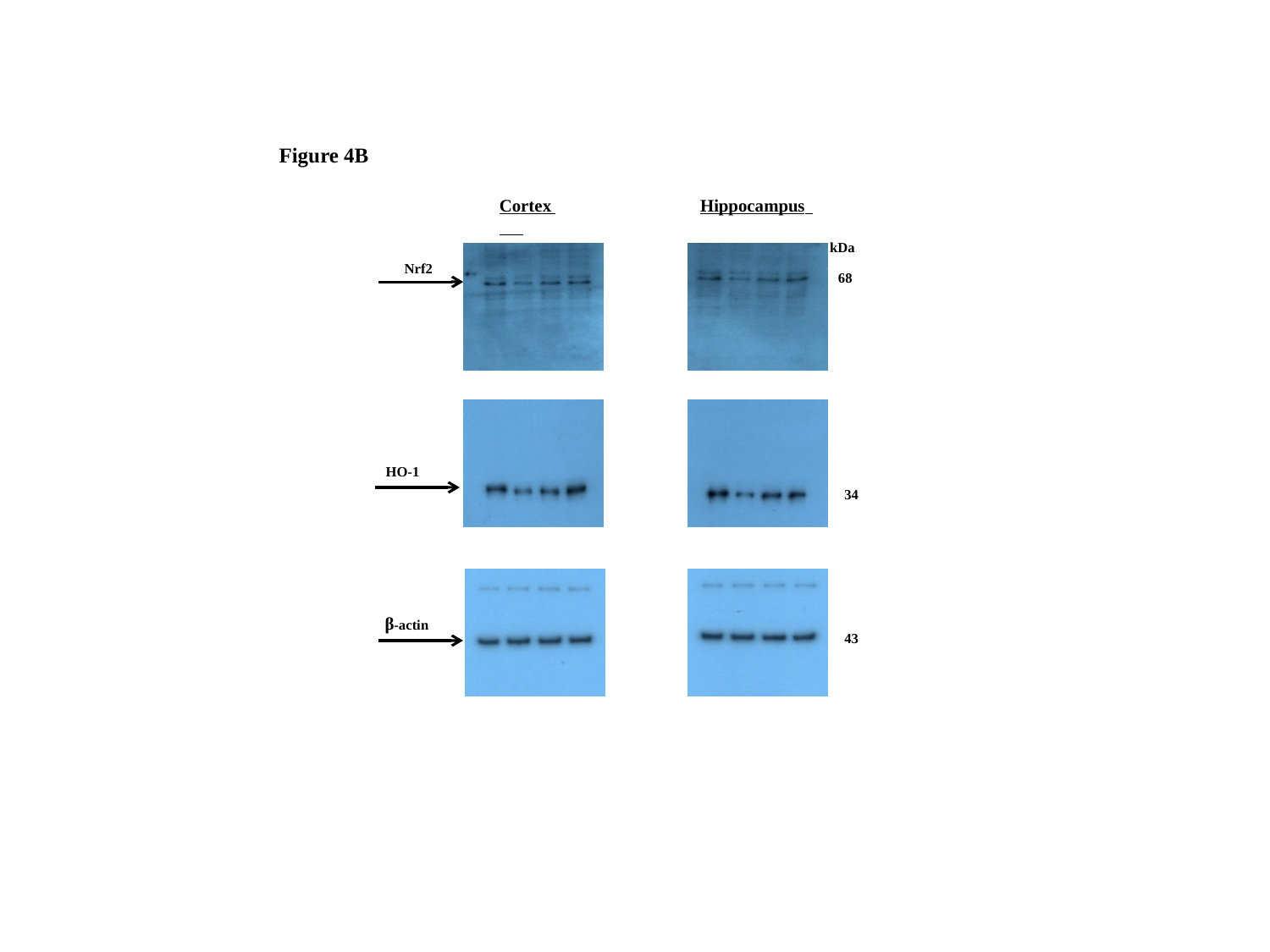

Figure 4B
Cortex
Hippocampus
kDa
 Nrf2
68
 HO-1
34
 β-actin
43

## Slide 5
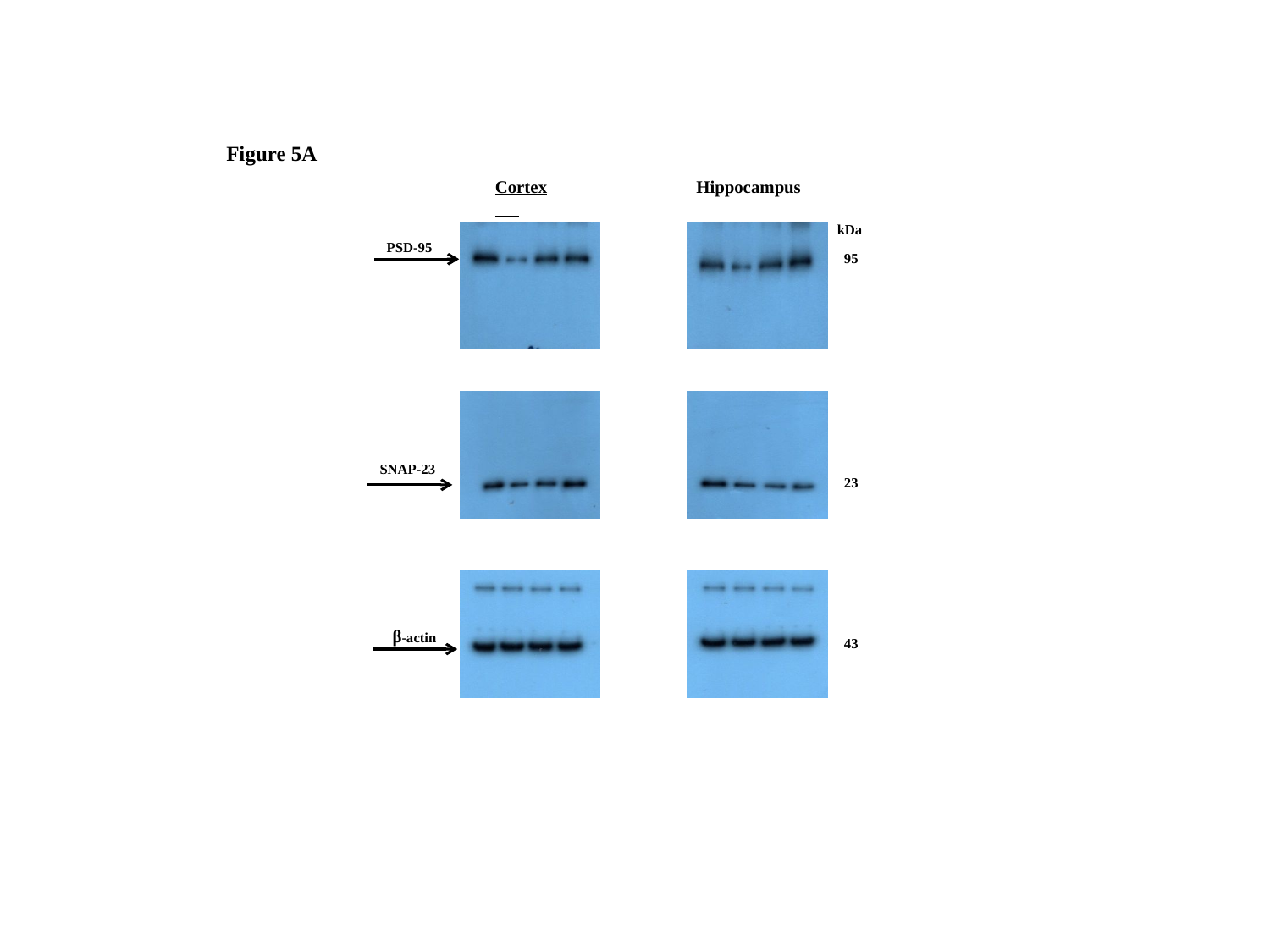

Figure 5A
Cortex
Hippocampus
kDa
PSD-95
95
SNAP-23
23
β-actin
43
